# Left ventricular fibro-fatty replacement in arrhythmogenic right ventricular dysplasia/cardiomyopathy: prevalence, patterns, and association with arrhythmias

**DOI:** 10.1186/s12968-020-00702-3

**Published:** 2021-05-20

**Authors:** Tarek Zghaib, Anneline S. J. M. Te Riele, Cynthia A. James, Neda Rastegar, Brittney Murray, Crystal Tichnell, Marc K. Halushka, David A. Bluemke, Harikrishna Tandri, Hugh Calkins, Ihab R. Kamel, Stefan Loy Zimmerman

**Affiliations:** 1 Division of Cardiology, Johns Hopkins University School of Medicine, Baltimore, MD USA; 2 Department of Cardiology, University Medical Center Utrecht, Utrecht, The Netherlands; 3 The Russell H. Morgan Department of Radiology and Radiological Sciences, Johns Hopkins University School of Medicine, 600 N. Wolfe St.; Halsted B180, Baltimore, MD USA; 4 Department of Pathology, Johns Hopkins University School of Medicine, Baltimore, MD USA; 5 Department of Radiology, University of Wisconsin School of Medicine and Public Health, Madison, WI USA

## Abstract

**Background:**

Left ventricular (LV) fibrofatty infiltration in arrhythmogenic right ventricular (RV) dysplasia/cardiomyopathy (ARVD/C) has been reported, however, detailed cardiovascular magnetic resonance (CMR) characteristics and association with outcomes are uncertain. We aim to describe LV findings on CMR in ARVD/C patients and their relationship with arrhythmic outcomes.

**Methods:**

CMR of 73 subjects with ARVD/C according to the 2010 Task Force Criteria (TFC) were analyzed for LV involvement, defined as ≥ 1 of the following features: LV wall motion abnormality, LV late gadolinium enhancement (LGE), LV fat infiltration, or LV ejection fraction (LVEF) < 50%. Ventricular volumes and function, regional wall motion abnormalities, and the presence of ventricular fat or fibrosis were recorded. Findings on CMR were correlated with arrhythmic outcomes.

**Results:**

Of the 73 subjects, 50.7% had CMR evidence for LV involvement. Proband status and advanced RV dysfunction were independently associated with LV abnormalities. The most common pattern of LV involvement was focal fatty infiltration in the sub-epicardium of the apicolateral LV with a “bite-like” pattern. LGE in the LV was found in the same distribution and most often had a linear appearance. LV involvement was more common with non-*PKP2* genetic mutation variants, regardless of proband status. Only RV structural disease on CMR (HR 3.47, 95% CI 1.13–10.70) and prior arrhythmia (HR 2.85, 95% CI 1.33–6.10) were independently associated with arrhythmic events.

**Conclusion:**

Among patients with 2010 TFC for ARVD/C, CMR evidence for LV abnormalities are seen in half of patients and typically manifest as fibrofatty infiltration in the subepicardium of the apicolateral wall and are not associated with arrhythmic outcomes.

**Supplementary Information:**

The online version contains supplementary material available at 10.1186/s12968-020-00702-3.

## Introduction

Arrhythmogenic right ventricular (RV) dysplasia/cardiomyopathy (ARVD/C) is a rare, inherited non-ischemic cardiomyopathy characterized predominantly by progressive fibrofatty RV replacement, ventricular arrhythmias, and an increased risk for sudden cardiac death. The pathogenesis of ARVD/C has been linked to defects in genes that encode structural proteins necessary for normal myocyte cell–cell adhesion, the most common of which in North-America is the gene for plakophilin-2 (*PKP2*) [1]. Varying degrees of left-ventricular (LV) involvement can be seen in ARVD/C, which can take the form of decreased LV systolic function, chamber dilation, wall motion abnormalities (WMA, defined as regional dyskinesia, akinesia, or dyssynchronous contraction), fat infiltration, and fibrosis [2–4].

Prior studies have shown a link between reduced LV function on echocardiography and either cardiac mortality or arrhythmias in ARVC [3, 5–7]. However, the significance of fibrofatty changes in the LV wall is uncertain. Both fat replacement and late-gadolinium enhancement (LGE), indicating fibrosis, have been reported in the LV on cardiovascular magnetic resonance imaging (CMR). However, detailed description of the typical patterns and distributions of these findings and their association with outcomes are lacking. The purpose of this study was to investigate the prevalence, patterns, and clinical significance, with respect to arrhythmic outcomes, of LV fibrofatty replacement on CMR in a large North American cohort of ARVD/C patients.

## Methods

### Study population

This is a retrospective study of consecutive subjects enrolled in our institutional ARVD/C registry from 2002–2012. Detailed information on phenotyping, definitions of clinical variables, family history collection, and follow-up has been previously described [8]. Briefly, this is an observational, prospective, cohort study initiated in 1999 with annual follow-up. All individuals provided written informed consent. The study protocol was Health Insurance Portability and Accountability Act compliant and approved by the Johns Hopkins Institutional Review Board. All registry subjects with a diagnosis of ARVD/C based on 2010 Task Force Criteria (TFC) [9] and available CMR imaging (n = 102) were reviewed; 29 patients were excluded because they were missing essential CMR sequences for evaluation of both fat and LGE (Additional file 1: Table S1). Genotype and variant adjudication are summarized in the Online Supplement. CMR studies were scored as major, minor, or no TFC based on qualitative and quantitative results as defined by the 2010 TFC. The total number of TFC points were tallied, with 2 points for each major criteria and 1 point for each minor criteria. Details on CMR imaging protocol are included in Additional file 1.

### CMR analysis

**Fig. 1:**
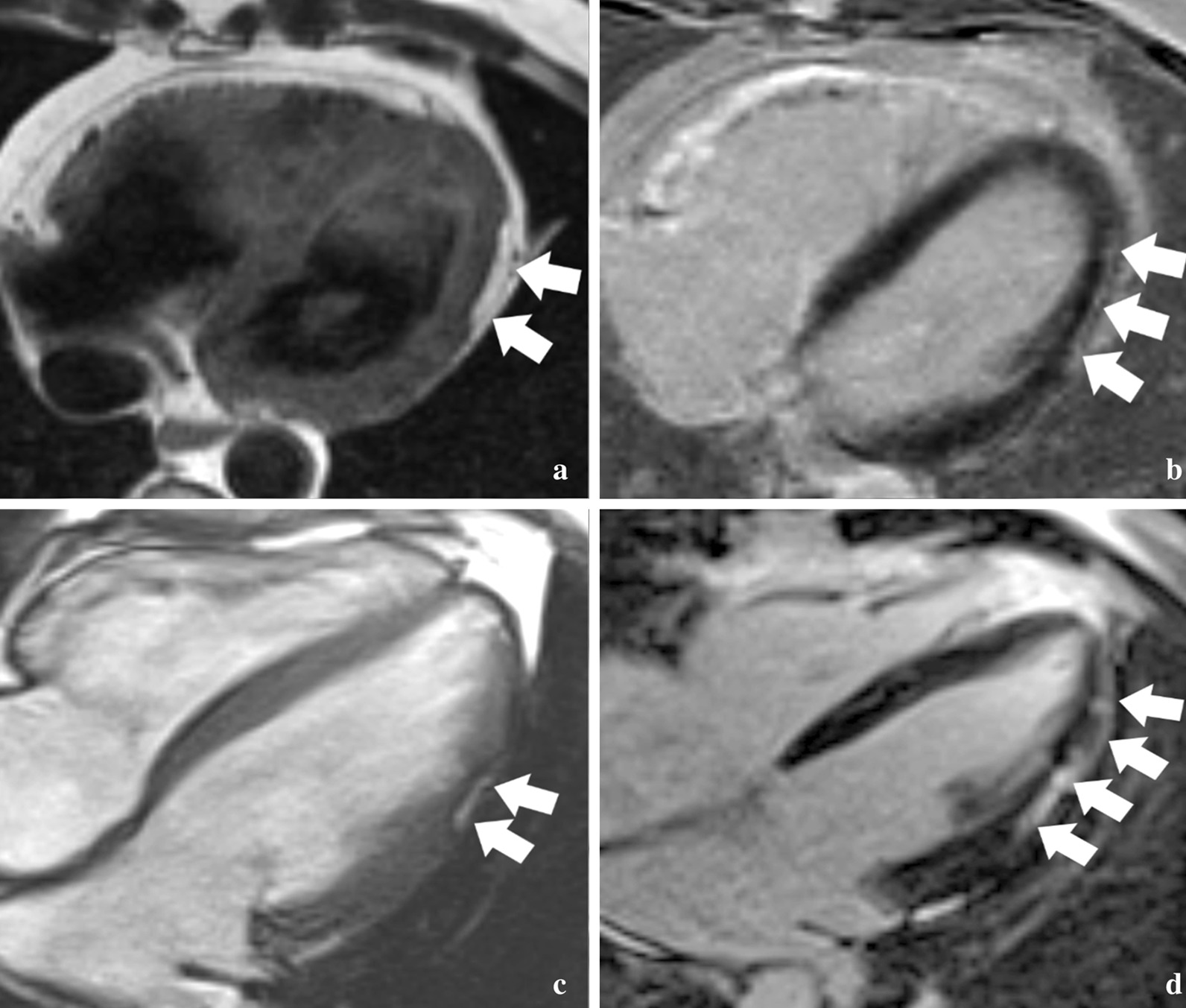
Patterns of left ventricular (LV) fat and fibrosis in arrhythmogenic right ventricular dysplasia/cardiomyopathy (ARVD/C). **a** Axial dark blood fast spin echo image obtained in a 46 year-old male proband with a PKP2 variant shows a typical bite-like pattern of epicardial fat infiltration in the apicolateral LV (arrows). **b** Axial late gadolinium enhancement (LGE) image in the same patient shows linear subepicardial enhancement suggesting fibrosis in the same location. **c** Axial balanced steady-state free precession cine image from a 28 year-old male proband with a PKP2 variant shows a typical “bite-like” pattern of LV fat infiltration on bright blood images, with the associated dark etching artifact between the fat and the myocardium. **d** Axial LGE image in the same patient shows more extensive enhancement indicating fibrosis in a predominantly linear subepicardial pattern along the lateral wall

LV and RV volumes and function were analyzed on dedicated software QMASS (Medis, Leiden, The Netherlands). Measurements were performed by a single reader with two years of CMR experience blinded to clinical information. Endocardial margins of LV and RV were manually contoured on end- systolic and end- diastolic images. Papillary muscles were included in the blood pool volume. Volumes were indexed to body surface area (BSA). End systolic volume index (ESVI), end diastolic volume index (EDVI), and ejection fraction (EF) for both ventricles were obtained. The ventricles were also assessed for WMA, fatty infiltration and LGE in consensus by two blinded CMR readers with ten and five years of experience, respectively. The location of cardiac abnormalities was recorded based on a 17 segment American Heart Association LV model and a 5 segment RV model previously described [10]. LV fat infiltration was identified by high signal within the LV myocardium on fast spin echo proton density/T1 weighted images or high signal with India-ink etching artifact on balanced steady-state free precession images as seen in Fig. 1 [11, 12]. In regions with suspected sub-epicardial fat infiltration, a contour deformity on the epicardial side of the LV wall with regional LV wall thinning were required to distinguish epicardial fat outside the LV from fatty infiltration involving the myocardium. LGE was identified by regions of increased signal relative to nulled myocardium. The presence of increased fast spin echo and LGE signal in the same segment were considered areas of overlap of fat and LGE. For fat infiltration and LGE, both the intra-mural location within the myocardium (transmural, subendocardial, mid-myocardial, sub-epicardial) and the pattern of involvement (patchy, linear, scalloped/bite-like), were recorded (Fig. 1). Patients with more than one intramural location or pattern of involvement were scored for both. LV involvement was defined as the presence of ≥ 1 of the following features: LV wall motion abnormality, LV-LGE, LV fat infiltration or LV-EF < 50%.

### Outcomes

The primary (arrhythmic) outcome was the time-to-occurrence (from baseline CMR) of sustained ventricular arrhythmia, defined as a composite of spontaneous sustained ventricular tachycardia (lasting ≥ 30 s at ≥ 100 bpm or with hemodynamic compromise requiring cardioversion), aborted sudden cardiac death, sudden cardiac death, or appropriate implantable cardioverter-defibrillator (ICD) intervention (shock or anti-tachycardia pacing) for a ventricular arrhythmia. Follow-up duration was calculated from the date of CMR to the date of reaching the endpoint as above or censoring, which was defined as death from any other cause, heart transplantation, or the most recent follow-up at which the endpoint could be ascertained through review of medical records. Outcomes were adjudicated at our Precision Medicine Center of Excellence in Arrhythmogenic Right Ventricular Cardiomyopathy and Complex Arrhythmias. In patients without an ICD, ventricular tachycardia (VT)/ventricular fibrillation (VF) outcome was adjudicated based on reviewing electrocardiograms (ECGs) and medical records; in patients with an ICD, the device-stored ECGs were reviewed for appropriateness of ICD therapy using standard device interrogation according to following definitions: VF or ventricular flutter was defined as an irregular or regular tachycardia with a mean cycle length ≤ 240 ms. VT was defined as a regular tachycardia arising from the ventricle with a cycle length > 240 and < 600 ms. Decisions regarding ICD programming were made by the managing cardiovascular specialist. When complete ICD interrogation information or ECG tracings were not available, interpretation by the referring electrophysiologist was used to classify arrhythmic events. In patients with multiple endpoints, time-to-outcome was measured at the first event.

### Statistical analysis

All continuous variables were reported as mean ± standard deviation and categorical variables as numbers (percentages). Continuous variables were compared using the independent Student t-test for normally distributed variables and Mann–Whitney U test for non-normally distributed variables. Categorical variables were compared using the Ficher’s exact test given sample size. Cumulative freedom from the composite arrhythmic outcome after the CMR exam was determined using multivariable Cox proportional-hazards models. All statistical analyses were performed using STATA (version 13, Stata Corporation, College Station, Texas, USA). A two-sided p-value of < 0.05 was considered statistically significant.

## Results

### Patient characteristics

The study population contained 73 patients with ARVD/C. Table 1 shows their clinical characteristics. Mean age of the study population was 34.2 ± 13.5 years, and 37 (50.7%) were male. A slight majority (n = 41, 56.2%) were probands, and the median number of TFC points was 6 (IQR 5–7). Of the study cohort, 35 (48%) carried an ARVD/C-associated pathogenic mutation, with *PKP2* the most common (n = 30) mutation. Additional file 1: Table S2 summarizes genetic characteristics of study participants with an ARVC/D-associated mutation.

**Table 1:** Demographic, clinical, and CMR data for the entire cohort, comparing subjects with and without left ventricular (LV) involvement at CMR

	All (n = 73)	No LV Involvement (n = 36)	LV Involvement (n = 37)	p-value
Demographics and clinical data				
Age (years)	34.2 ± 13.5	34.8 ± 15.7	33.6 ± 11.2	0.81
Male sex (%)	37 (50.7)	16 (44.4)	21 (56.8)	0.35
Mutation positive (%)	35 (48.0)	17 (47.2)	18 (48.7)	1.00
PKP2 (%)	30 (41.1)	17 (47.2)	13 (35.1)	0.39
DSP (%)	1 (1.4)	0 (0)	1 (2.7)	
DSC2 (%)	0 (0)	0 (0)	0 (0)	
DSG2 (%)	1 (1.4)	0 (0)	1 (2.7)	
PLN (%)	1 (1.4)	0 (0)	1 (2.7)	
Compound^a^ (%)	2 (2.7)	0 (0)	2 (5.4)	
Proband (%)	41 (56.2)	14 (39.0)	27 (73.0)	0.005
TFC points	6 (IQR 5–7)	5 (IQR 4–6.5)	7 (IQR 5–8)	0.001
CMR findings				
RVEDVI (mL/m^2^)	106.7 ± 31.0	101.5 ± 26.1	111.9 ± 34.6	0.19
RVEF (%)	41.7 ± 10.0	46.3 ± 8.7	37.2 ± 9.2	< 0.001
LVEDVI (mL/m^2^)	89.3 ± 19.8	87.9 ± 17.8	90.5 ± 21.6	0.45
LVEF (%)	54.1 ± 6.6	57.9 ± 4.3	50.3 ± 6.5	< 0.001
RV-LGE (% with any RV LGE)	13 (17.8)	6 (16.7)	7 (18.9)	1.00
RV Fat (% with any RV fat)	22 (30.1)	9 (25.0)	13 (35.1)	0.45
RV-WMA (% with any RV WMA)	54 (74.0)	20 (55.6)	34 (91.9)	< 0.001

*LV* left ventricle, *TFC* task force criteria, *CMR* cardiovascular magnetic resonance imaging, *RVEDVI* right ventricular end-diastolic volume indexed to body surface area, *RVEF* right ventricular ejection fraction, *LVEDVI* LV end-diastolic volume indexed to body surface area, *LVEF* LV ejection fraction, *RV-LGE* right ventricular late gadolinium enhancement, *RV fat* right ventricular fat, *RV-WMA* right ventricular wall motion abnormality

^a^One patient had both a *PKP2* and a *DSP* mutation. A second patient had two different *DSG2* mutations in trans (compound heterozygous)

### Prevalence of LV involvement on CMR

Among the overall population, 37 (50.7%) patients had LV involvement. As seen in Table 1, patients with and without LV involvement were similar in age (p = 0.81) and sex (p = 0.35). Those with LV involvement were more likely to be probands and had significantly higher number of TFC points (5 [IQR 4–6.5] vs. 7 [IQR 5–8], p = 0.001), lower RV-EF (36.9 ± 9.4% vs. 45.2 ± 9.0%, p < 0.001) and more prevalent RV-WMAs (91.9% vs. 55.6%, p < 0.001). There was no difference in the likelihood of carrying an ARVD/C-associated genotype variant between patients with and without LV involvement, even after controlling for proband status.

### Patterns of LV fat and LGE

**Fig. 2:**
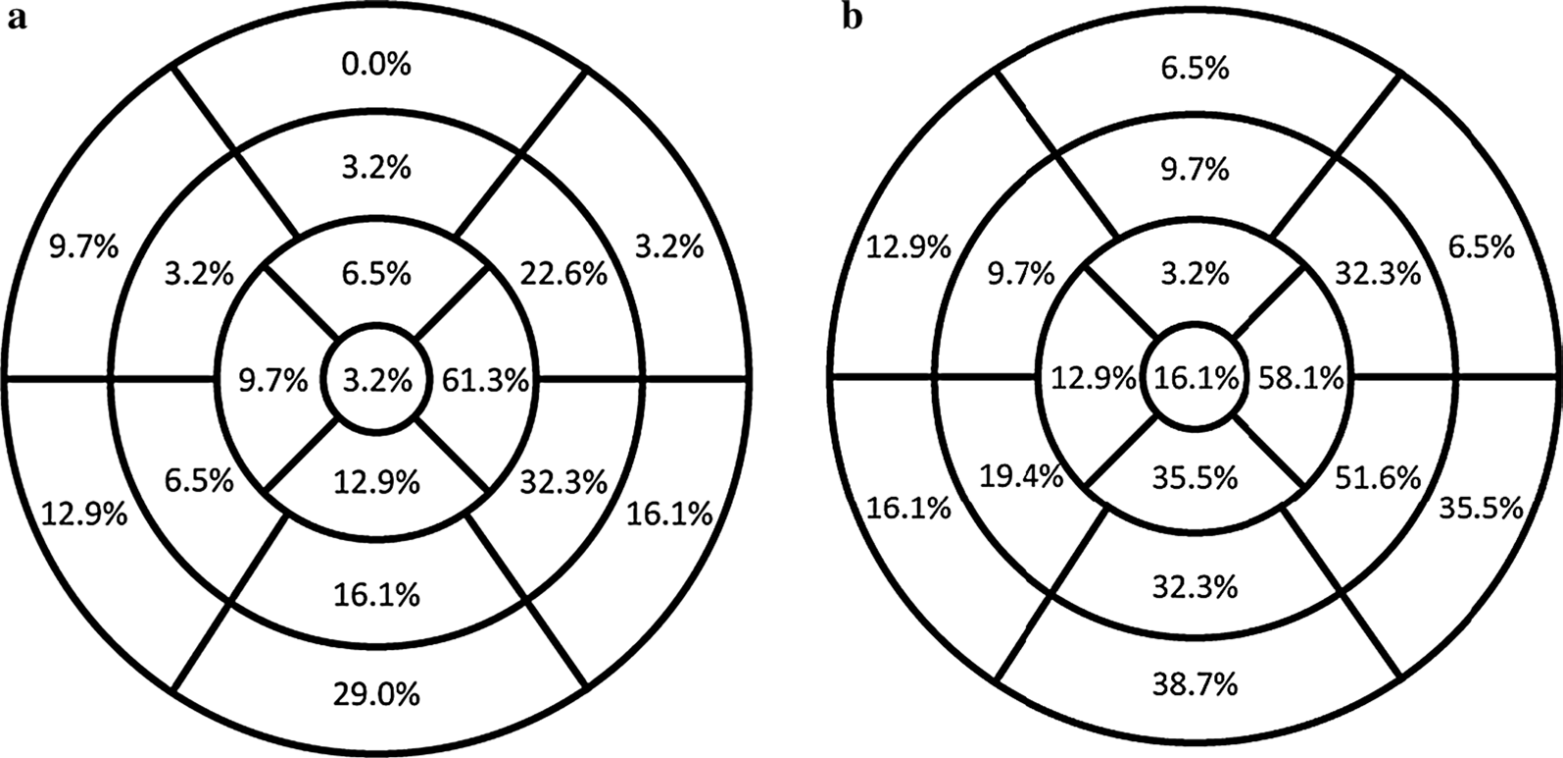
Bullseye plot of the distribution of left ventricular (LV) fat (A) and late gadolinium enhancement (LGE) (B) by American Heart Association segments among the 31 ARVD/C subjects with LV abnormalities at CMR. As seen in the figures, Fat and LGE were most commonly localized in the apico-lateral, infero-basal and lateral walls of the LV

Fat and LGE were most often localized in the apico-lateral, infero-basal and lateral walls of the LV (Fig. 2). LGE was typically sub-epicardial and linear in location and pattern, whereas fat was sub-epicardial but often had a scalloped/bite-like pattern (Fig. 1 and Table 2). The mid-myocardium was involved only half as frequently as the sub-epicardium with both fat and LGE, while the sub-endocardium was never involved. LV segments with isolated LGE (70/527, 13.2%) were more frequent than isolated fat (23/527, 4.3%), and LGE-fat overlap occurred in 53/527 (10.1%) of segments in patients with LV involvement.

**Table 2:** Typical patterns of left ventricular abnormalities among 37 subjects with LV involvement

	LGE	Fat	WMA	Low EF
Subjects (%)	27 (73.0)	25 (67.6)	9 (24.0)	18 (48.7)
Most frequent American Heart Association segment involved	Apico-lateral (n = 18)	Apico-lateral (n = 19)	Mid-inferolateral (n = 6)	–
Myocardium involved				
Sub-endocarium (%)	0 (0)	0 (0)	–	–
Mid-myocardium (%)	14 (45.2)	8 (25.8)	–	–
Sub-epicardium (%)	24 (77.4)	17 (54.8)	–	–
Trans-mural (%)	2 (6.5)	0 (0)	–	–
Pattern				
Linear (%)	21 (67.7)	9 (29.0)	–	–
Patchy (%)	6 (19.4)	2 (6.5)	–	–
Bite/scalloped (%)	5 (16.1)	19 (61.3)	–	–

*EF* ejection fraction, *LGE* late gadolinium enhancement, *WMA* wall motion abnormality

### Genotype-specific LV changes

As seen in Table 3, there were no differences in RV size or function on CMR between all 3 groups (no mutation vs. *PKP2* vs. non-*PKP2* variants), but patients in the latter group had lower LVEF, more prevalent LV-LGE as well as LV-WMAs. With regards to fat patterns, 7/14 patients with “bite-like” fatty infiltration carried a mutation, all were *PKP2* mutations. *O*nly two patients had isolated LV involvement (without RV abnormalities), one with an extra-desmosomal mutation in the phospholamban (*PLN*) gene, and the other with a desmoplakin (*DSP*) mutation. Interestingly, both of these patients had extensive LV-LGE but no LV fat. Characteristics of the two patients are summarized in Additional file 1: Table S3.

**Table 3:** Demographic, clinical, and CMR data for the entire cohort, comparing subjects with and without PKP2 variant

	All (n = 73)	No mutation (n = 38)	PKP2 mutation (n = 30)	Other mutations^a^ (n = 5)	p-value**
Demographics and clinical data					
Age (years)	34.2 ± 13.5	34.8 ± 13.6	33.7 ± 14.0	33.1 ± 12.8	0.934
Male sex (%)	37 (50.7)	19 (50.0)	18 (60.0)	0 (0)	0.045
Proband (%)	41 (56.2)	25 (65.8)	13 (43.3)	3 (60.0)	0.177
VT prior to CMR	29 (39.7)	16 (42.1)	12 (40.0)	1 (20.0)	0.637
VT during follow-up	37 (50.7)	20 (52.6)	16 (53.3)	1 (20.0)	0.363
CMR findings					
RVEDVI (mL/m^2^)	106.7 ± 31.0	111.8 ± 30.9	104.0 ± 30.7	84.8 ± 25.7	0.152
RVEF (%)	41.7 ± 10.0	41.6 ± 9.8	42.0 ± 10.7	40.0 ± 8.9	0.918
LVEDVI (mL/m^2^)	89.3 ± 19.8	93.2 ± 23.1	85.0 ± 14.5	87.2 ± 20.0	0.245
LVEF (%)	54.1 ± 6.6	54.7 ± 6.7	54.5 ± 5.9	47.3 ± 7.8	0.057
LV-LGE (%)	27 (37.0)	15 (39.5)	7 (23.3)	5 (100.0)	0.004
LV Fat (%)	25 (34.3)	14 (36.8)	9 (30.0)	2 (40.0)	0.808
LV-WMA (%)	9 (12.3)	3 (7.9)	2 (6.7)	4 (80.0)	< 0.001
Low LV-EF (< 50%) (%)	18 (24.7)	9 (23.7)	6 (20.0)	3 (60.0)	0.155

*LV* left ventricle, *TFC* Task Force Criteria, *RV-EDVI* right ventricular end-diastolic volume indexed to body surface area, *RVEF* right ventricular ejection fraction, *LV-EDVI* LV end-diastolic volume indexed to body surface area, *LVEF* LV ejection fraction, *RV-LGE* right ventricular late gadolinium enhancement, *RV fat* right ventricular fat, *RV-WMA* right ventricular wall motion abnormality, *VT* ventricular tachycardia

**p-value derived from ANOVA test. No significant difference was found between the No Mutation and PKP2 Mutation groups on Rank-Sum or Ficher’s Exact tests for all the above demographic and CMR variables

^a^Mutations include *DSP*, *DSG2*, *DSC2, PLN*, and compound non-PKP2 mutations. One patient had both a *PKP2* and a *DSP* mutation and was included in PKP2 + ve group

### Arrhythmic outcomes

Patients were followed for a mean 4.6 ± 2.9 years after baseline CMR. The majority (57/73, 78.1%) of patients had an ICD implanted during the follow-up period. The arrhythmic outcome was reached by 37 patients, all of whom underwent ICD implantation during the follow-up period. The most common outcome was ICD therapy which occurred in 25/73 (34.2%), followed by sustained VT (11/73, 15.1%), and a single life-vest shock (2.7%). Mean cycle length was available in 25 patients with arrhythmic outcomes and was 277.8 ± 56.5 ms. No deaths occurred during follow up.

**Fig. 3:**
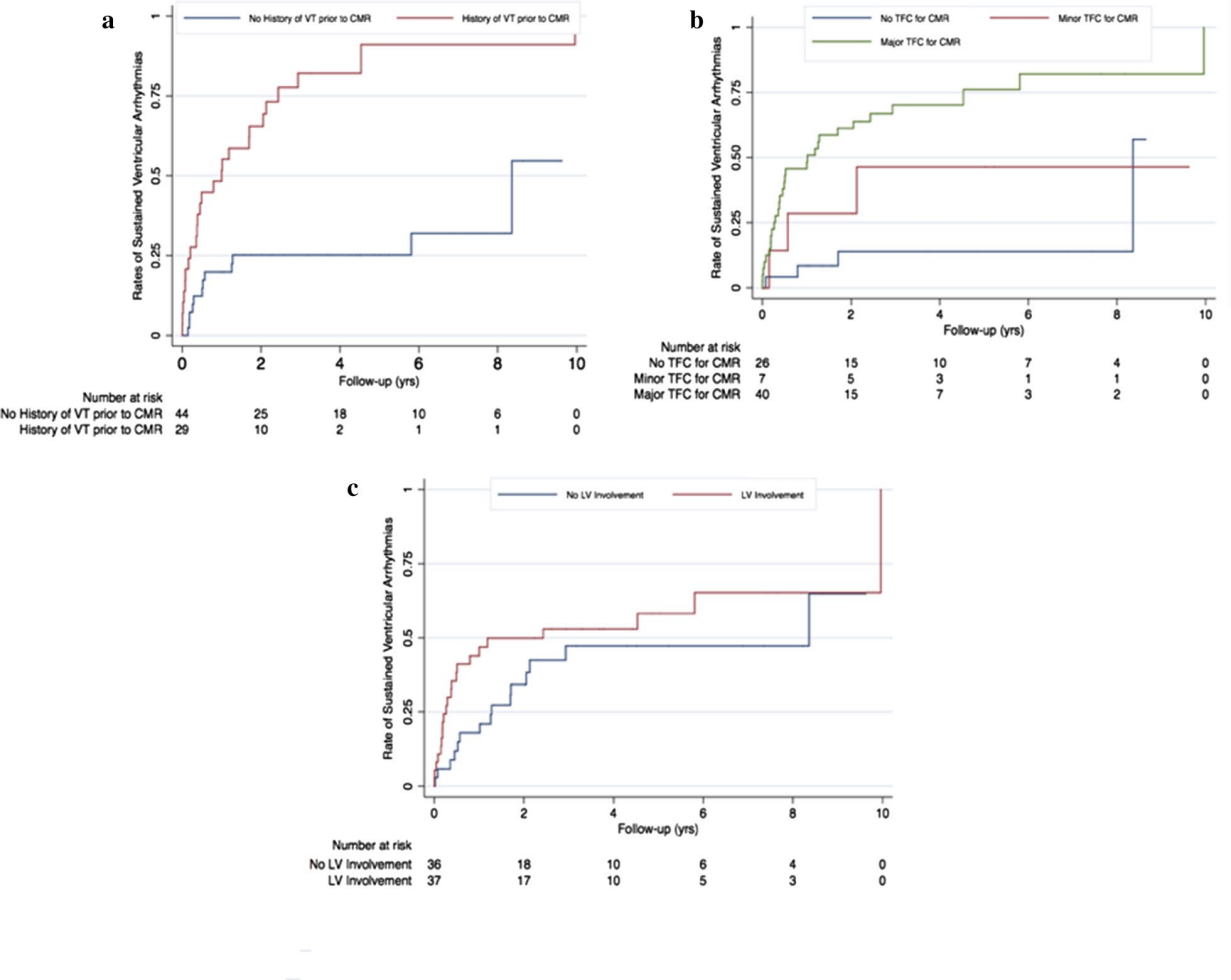
Kaplan–Meier survival curves for sustained ventricular arrhythmias in the study population stratified according to **a** history of sustained ventricular arrhythmias; **b** CMR Task Force Criteria (TFC) status (as a marker of right ventricular (RV) disease severity), and **c** LV Involvement on CMR. Survival curves illustrate the findings of our multivariate analysis that a history of prior arrhythmia as well as advanced RV—rather than LV—structural disease at baseline CMR independently portray increased risk of arrhythmic events during follow-up

Patients with arrhythmic outcomes were more often males, and had a history of VT (Table 4). No genotype differences were seen between patients with vs. without arrhythmias on follow-up. On CMR analysis, RV- and LVEDVI were greater and RV-EF lower in patients with arrhythmias, while LVEF was similar between the two groups. LV fat, RV-WMAs and RV-LGE were significantly more common in patients with arrhythmias, whereas RV fat and LV-WMAs were not. LV-LGE was more common in patients with arrhythmias although this did not achieve statistical significance. As such, patients experiencing arrhythmias were significantly more likely to fulfill major CMR-TFC compared to those without arrhythmias (92.2% vs. 35.9%, p < 0.001). On univariate Cox proportional-hazards analysis, male sex, history of prior sustained ventricular arrhythmia, CMR-TFC status (a marker of RV disease severity), and LV-LGE were associated with ventricular arrhythmias on follow-up. However, on multivariate analysis shown in Table 5, only a history of prior sustained ventricular arrhythmias and CMR-TFC were significantly associated with the outcome. Kaplan–Meier curves for arrhythmic outcomes stratified by CMR-TFC status, VT history at baseline, and LV involvement on CMR are shown in Fig. 3.

**Table 4:** Comparison of demographic and CMR data for subjects with and without ventricular arrhythmias during follow-up

	Ventricular arrhythmia (n = 37)	No ventricular arrhythmia (n = 36)	p-value
Demographics and clinical			
Age (years)	34.3 ± 13.6	34.2 ± 13.6	0.85
Male sex (%)	25 (67.6)	12 (33.3)	0.005
Gene positive (%)	17 (46.0)	18 (50.0)	0.82
VT prior to CMR (%)	25 (67.6)	4 (11.1)	< 0.001
RV CMR findings			
RV WMA (%)	34 (91.9)	20 (55.6)	< 0.001
RV Fat (%)	14 (37.8)	8 (22.2)	0.20
RV LGE (%)	11 (29.7)	2 (5.6)	0.012
RVEDVI (mL/m^2^)	119.7 ± 30.2	93.4 ± 25.9	< 0.001
RVEF (%)	37.5 ± 7.8	46.0 ± 10.3	< 0.001
LV CMR findings			
LV WMA (%)	5 (13.5)	4 (11.1)	1.000
LV fat (%)	17 (45.9)	8 (22.2)	0.048
LV fat in a bite pattern (%)	12 (32.4)	2 (5.6)	0.006
LV LGE (%)	18 (48.7)	9 (25.0)	0.052
LV fat and/or LGE (%)	20 (54.1)	11 (30.6)	0.059
LVEDVI (mL/m^2^)	93.8 ± 21.7	84.7 ± 16.6	0.011
LVEF (%)	53.8 ± 6.8	54.4 ± 6.5	0.45
Low LVEF (< 50%) (%)	10 (27.0)	8 (22.2)	0.79
Any LV Involvement	22 (59.5)	15 (41.7)	0.16
CMR task force criteria			
Any TFC for CMR (%)	34 (92.2)	13 (35.9)	< 0.001
TFC for CMR major (%)	30 (81.1)	10 (27.8)	< 0.001
TFC for CMR minor (%)	4 (11.1)	3 (8.1)	1.000
Total TFC Score	7 (IQR 6–8)	5 (IQR 5–6)	< 0.001

*RV* right ventricle, *VT* ventricular tachycardia, *LV* left ventricle, *WMA* wall motion abnormality, *LGE* late gadolinium enhancement, *RVEDVI* right ventricular end-diastolic volume indexed to body surface area, *RVEF* right ventricular ejection fraction, *LVEDVI* LV end-diastolic volume indexed to body surface area, *LVEF* LV ejection fraction, *TFC* task force criteria

**Table 5:** Univariate and multivariate Cox regression analysis of the relationship between ventricular tachycardia events in follow-up and demographic and CMR findings

	Univariate		Multivariate	
	HR (95% CI)	p-value	HR (95% CI)	p-value
Age	0.99 (0.97–1.02)	0.491	–	–
Male sex	2.20 (1.09–4.41)	0.027	–	–
Prior VT prior to CMR	4.54 (2.24–9.22)	< 0.001	2.85 (1.33–6.10)	0.007
TFC for CMR (major or minor)	5.85 (2.06–16.59)	0.001	3.47 (1.13–10.70)	0.030
LV Fat	1.75 (0.90–3.38)	0.098	–	–
LV Fat in a bite pattern	1.99 (0.97–4.08)	0.059	–	–
LV LGE	1.99 (1.03–3.84)	0.040	–	–
Low LVEF (< 50%)	0.99 (0.47–2.11)	0.979	–	–

*TFC* task force criteria, *LV* left ventricle, *WMA* wall motion abnormality, *LGE* late gadolinium enhancement

We subdivided patients with preserved LVEF according to the presence or absence of tissue abnormalities on CMR. As seen in Supplemental Table 4, patients with detectable LV fibrofatty infiltrates on CMR had higher TFC score, and were more likely to be probands. The Kaplan–Meier Survival curve (Additional file 1: Figure S1) shows that they had higher rates of VT throughout the follow-up period without achieving statistical significance (log-rank p = 0.08).

## Discussion

In this retrospective analysis of LV abnormalities in a large cohort of North American patients with ARVD/C we describe several important results. First, regional abnormalities in the LV, including fatty infiltration and fibrosis, were found in 51% of ARVD/C patients, were most often localized to the apico-lateral portion of the LV in a sub-epicardial distribution, were associated with more advanced disease, and were most prevalent in patients with non-*PKP2* ARVD/C-associated variants. Second, these LV abnormalities had no significant independent association with arrhythmic events after adjusting for clinical variables and extent of RV disease. Third, the strongest risk factors for future arrhythmic events in this cohort were the presence of CMR evidence for RV structural abnormalities to meet CMR TFC and a history of ventricular arrhythmia.

### LV fibrofatty infiltration—prevalence and patterns

The presence of fibrofatty infiltration in the LV of ARVD/C patients is a well-known phenomenon, initially described in the pathology literature from autopsy specimens. In the largest, autopsy-based multicenter study of 202 ARVD/C hearts after sudden cardiac death, there was histopathological LV involvement in 87% of cases, of which 17% had isolated LV changes [13]. Similarly, in another study of 42 ARVD/C hearts, LV fibrofatty changes were identified in 76% of cases, with a predilection for involvement of the sub-epicardial region of the LV wall [2]. This high frequency of LV abnormalities has led to the suggestion that the more generic and encompassing term, “arrhythmogenic cardiomyopathy” be used to describe this disease [1]. However, current TFC diagnostic criteria for CMR rely solely right-sided abnormalities, and as such we have continued to use the term ARVD/C for these patients. In the current study, we found LV-LGE in 37% of our cohort. In these cases, LV-LGE most often had a linear, sub-epicardial distribution, or, less frequently, a patchy, midmyocardial pattern. Previous LGE-CMR studies have non-invasively confirmed the sub-epicardial-predominant distribution of fibrosis in ARVD/C, with a variable prevalence in patients ranging from 14–84% [14–16]. Discrepancy in prevalence between CMR-based and autopsy-based studies may be due to lower sensitivity and resolution of CMR to detect microscopic fibrofatty infiltration of the cardiac muscle, as well as a selection bias in autopsy studies towards patients with more advanced disease who died of sudden cardiac death.

In most cases in our present study, fat and LGE were seen in the same segment, suggesting the process of fibrofatty replacement as reported by autopsy studies [2, 17, 18]. However, in some cases without regional overlap, fat identify and fibrosis were identified as separate entities. In general, LV fat on CMR has received less attention than LGE, being reported in a relatively small number of studies [16, 19]. Our study shows that similar to LGE, LV fat is most often found in the apico-lateral wall with a sub-epicardial distribution, in accord with prior reports [19, 20]. Interestingly, we found fat infiltration in the LV most often showed a unique scalloped or bite-like pattern. CMR patterns of LV-LGE and LV fat may have important relevance to the diagnosis of ARVD/C. Sarcoidosis is an important mimic of ARVD/C and can have significant overlap in clinical presentation, electrical findings, and extent of structural heart disease [21]. Indeed, the pattern of LV-LGE we have observed in this cohort (sub-epicardial location, linear pattern) has substantial overlap with the imaging pattern expected for sarcoidosis. To further add to potential diagnostic confusion, it is well known that in addition to the LV, the RV is frequently involved in sarcoidosis, manifested by both systolic dysfunction and LGE, and indeed is an important negative prognosticator for these patients [22]. In contradistinction to LGE, the bite-like pattern of fat infiltration we describe has never been reported for any disease besides ARVD/C, and as such, we believe it may have utility in distinguishing ARVD/C from sarcoidosis and other mimics. It is important to contrast this concept of LV fat at CMR in ARVD/C, which we hypothesize could prove a specific disease marker, with RV fat at CMR, which has been more thoroughly studied and is known to lack diagnostic sensitivity and specificity [23, 24], due to both overlap with non-ARVD/C entities [25] and challenges with imaging of the thin RV wall [26].

### LV fibrofatty infiltration—genotype–phenotype determination

Previous studies have shown that certain ARVD/C variants are strongly associated with LV fibrofatty changes, regardless of proband status [27–29]. In mutation positive ARVD/C, *DSP* carriers are associated with four-fold higher likelihood of sudden cardiac death, LV dysfunction, and heart failure compared to *PKP2* carriers [27]. Similarly, *PLN*, *DSP, DSG2* and *DSC2* carriers were found to have higher rates of LV dysfunction compared to *PKP2* [27, 29, 30]. Similarly, in a study by Groeneweg et al., non-desmosomal *PLN* variants were more likely to have LV involvement compared with desmosomal mutation carriers [31]. In a study of 289 ARVC/D patients, Gilotra et al. did not find an association between odds of heart failure and presence of a genetic mutation; interestingly, all patients with more than one mutation in this study had CHF, and the *DSP* variant was more common in patients with LV dysfunction [5]. Despite the small number of non-*PKP2* variants in our cohort, our findings align with prior reports such that non-*PKP2* variants carriers had a higher likelihood of exhibiting LV disease such as WMAs, low LVEF, LV-LGE and LV-fat (Table 3). Interestingly, in 3 patients, a predominantly fibrotic pattern was seen with isolated LV-LGE and no LV fat. All were positive for gene variants that have been associated with the “left-sided” ARVD/C phenotype (*PLN, DSP, DSG2*) [28, 29, 32]. These findings further reinforce the concept of genotype–phenotype correlations for LV involvement in ARVD/C, with subepicardial fibrofatty LV changes seen among *PKP2* mutation carriers and diffuse LV fibrosis and dysfunction among *DSP* mutation carriers [33].

### Arrhythmic outcomes

LV abnormalities have been inconsistently associated with adverse outcomes in ARVD/C. Several European groups have shown a strong, independent association of LV dysfunction with cardiac death or life-threatening arrhythmias [2, 3, 7]. However, other studies of North American populations have not replicated these findings [8, 31, 34]. In 365 patients with definite ARVC/D from The Johns Hopkins ARVD/C Registry, LV dysfunction was not found to be a significant predictor of sustained VT/VF during follow-up [31]. In the largest multicenter and multinational cohort study to date (n = 976 patients), Cadrin-Tourigny et al. retrospectively demonstrated that LV EF on echocardiography is not a significant predictor of sustained ventricular arrhythmias after adjusting for RV dysfunction [35]. Similar to these findings, we found no difference in LV systolic function between patients with or without arrhythmic events at follow-up. Genotypic differences between European and North American ARVD/C populations may be driving these disparate findings with increased prevalence of patients with *DSP* variants in European cohorts [36]. These results reinforce a growing body of evidence suggesting that RV structural disease on CMR is of critical importance when assessing arrhythmic risk. In a recent study of ARVD/C mutation carriers, arrhythmic events only occurred in patients with structural disease in the RV, whereas those with isolated electrical abnormalities did not experience arrhythmic events at follow-up [37]. In another study from Deac et al. RV abnormalities identified on CMR had significant prognostic value; with a negative predictive value for future of events of 98.8% for a normal CMR study [38].

### Pathophysiology of LV abnormalities in ARVC/D

The significance of these regional abnormalities of the LV wall, independent of global LV dysfunction, has not been previously described in imaging studies. A prior study has also reported inducible LV-based VTs during electrophysiological studies performed in ARVD/C patients. The VTs localized to the LV posterolateral wall, corresponding to the most common location of involvement of LV fat and fibrosis on CMR [10]. These findings raise the question of whether the identification of LV involvement by CMR has prognostic significance for determining future arrhythmic risk. Interestingly, in this current study we show that, although fibrosis and fat in the LV wall were seen more often in patients with arrhythmic events, there was no association with arrhythmic outcomes at follow-up after controlling for RV disease severity and history of arrhythmias prior to CMR. Pathophysiologically, it is important to distinguish between extensive LV scar involving a large portion of the LV and leading to heart failure, as opposed to localized and focal fibrofatty replacement leading to abnormal electrical conduction and sustained ventricular arrhythmias. On subgroup analysis, participants with preserved LV-EF and tissue abnormalities had higher rates of VT throughout follow-up compared to those with preserved LV-EF without tissue abnormalities, albeit not reaching statistical significance. As such, further and larger studies are needed to compare risks of life-threatening arrhythmias independently incurred by focal LV fibrofatty infiltration in patients with ARVD/C independent of global systolic dysfunction.

### Limitations

Our study has several limitations. First, this is a retrospective study of a cohort of ARVD/C patients from a tertiary care center, which may result in referral bias, however, this is unavoidable given the rarity of this disease. Second, given the known regional/ethnic variability in ARVD/C-associated pathogenic mutations leading to unbalanced predominance of PKP2-positive patients in our North-American cohort, we are limited in our ability to draw statistically significant conclusions with regards to any genetic association in terms of LV involvement. Third, we do not have pathologic proof the LGE identified at CMR represents myocardial fibrosis, although this concept has been supported by a large body of literature in ischemic and non-ischemic cardiomyopathies [3]. Fourth, fat infiltration may result in increased signal on LGE-based CMR imaging, therefore some cases with presumed overlap of fat and fibrosis could represent predominantly fat infiltration. Fifth, we did not report the prevalence of focal "crinkling" of the RV wall (also known as “accordion sign”). This finding was described in mutation positive, asymptomatic first degree relatives of ARVC patients, highlighting the utility of this finding as a potential early sign of the disease [40]. The utility of this minor finding in probands with advanced global/regional structural disease is very limited, and the reproducibility of identifying the accordion sign in the presence of moderate to severe myocardial dilation, aneurysms and wall motion abnormalities is unknown. Finally, given that we have not performed endomyocardial biopsies we cannot exclude the possibility of prior subclinical myocarditis to explain prevalent LV tissue abnormalities.

## Conclusions

In a North American cohort of ARVD/C patients, LV involvement was found in half of the patients. LV disease is most often characterized by fibrofatty infiltration in the sub-epicardium of the apico-lateral wall. Fat infiltration was often seen in a bite-like pattern, which may be a unique marker of ARVD/C. Although LV abnormalities were more common in patients experiencing arrhythmic events, there was no significant independent association with outcomes on multivariable analysis. History of prior arrhythmic event and extent of RV disease are the only significant predictors of arrhythmic events after CMR.

## Supplementary information

**Additional file 1.** Cardiac MRI protocol and supplemental tables and figures.

## Data Availability

The datasets used and/or analysed during the current study are available from the corresponding author on reasonable request.

## Abbreviations

ARVD/C: Arrhythmogenic right ventricular dysplasia/cardiomyopathy
BSA: Body surface area
CMR: Cardiovascular magnetic resonance imaging
*DSG2*: Desmoglein-2
*DSP*: Desmoplakin
EDVI: End diastolic volume index
EF: Ejection fraction
ECG: Electrocardiogram
ESVI: End systolic volume index
ICD: Implantable cardioverter defibrillator
LGE: Late gadolinium enhancement
LV: Left ventricle/left ventricular
LVEF: Left ventricular ejection fraction
*PKP2*: Plakophilin-2
*PLN*: Phospholamban
RV: Right ventricle/right ventricular
RVEF: Right ventricular ejection fraction
TFC: Task Force Criteria
VF: Ventricular fibrillation
VT: Ventricular tachycardia
WMA: Wall motion abnormality(s)
