# Supplementary material for: Left ventricular fibro-fatty replacement in arrhythmogenic right ventricular dysplasia/cardiomyopathy: prevalence, patterns, and association with arrhythmias
Source: J Cardiovasc Magn Reson. 2021 May 20;23:58. doi: 10.1186/s12968-020-00702-3 (PMC8135158; doi:10.1186/s12968-020-00702-3)
Supplement: Supplementary file 1 — Additional file 1. Cardiac MRI protocol and supplemental tables and figures. [file 12968_2020_702_MOESM1_ESM.docx]

**Additional file 1**

Methods

*CMR Imaging Protocol*

Studies performed at the authors’ institution (n=60) were obtained on 1.5-T scanners (CV/I, GE Medical Systems, Waukesha, Wisconsin, or Siemens, Avanto, Erlangen, Germany. Studies from outside centers (CV/I, GE Medical Systems, Waukesha, Wisconsin, or Siemens, Avanto, Erlangen, Germany) (n = 13) were also all performed at 1.5T. For inclusion, studies must have included a minimum of double-inversion recovery fast spin echo (FSE) sequences, cine functional images in the long and short-axis planes for quantification of volumes and ejection fraction (EF), and post-contrast LGE images. Research review of CMRs was not used to establish ARVD/C diagnosis, which was strictly based on 2010 global TFC at the time of clinical diagnosis. Due to the rarity of the ARVD/C disease, some of the members of this patient cohort have been included in previous studies (1–4).

*Genotype and Variant Adjudication*

Genotyping was performed as previously described (5). Probands had all undergone sequencing of the desmosomal genes, *PLN*, and *TMEM43* at a minimum. Affected family members typically were tested only for the variant(s) identified in their family. Variants included in this study were (re)adjudicated in November 2018 according to the American College of Medical Genetics and Genomics/Association for Molecular Pathology guidelines for variant interpretation with adaptations recommended by the ClinGen Inherited Cardiomyopathy Expert Panel (6, 7). Only variants classified as Pathogenic or Likely Pathogenic (class V or IV) per these criteria were considered mutations in this study.

Additional file References

1. Rastegar N, Zimmerman SL, Te Riele ASJM, et al. Spectrum of Biventricular Involvement on CMR among Carriers of ARVD/C-Associated Mutations. JACC Cardiovasc. Imaging 2015;8:863–864.

2. Te Riele ASJM, James CA, Philips B, et al. Mutation-positive arrhythmogenic right ventricular dysplasia/ cardiomyopathy: The triangle of dysplasia displaced. J. Cardiovasc. Electrophysiol. 2013;24:1311–1320.

3. Madhavan S, Calkins H, Kamel IR, et al. Incremental Value of Cardiac Magnetic Resonance Imaging in Arrhythmic Risk Stratification of Arrhythmogenic Right Ventricular Dysplasia/Cardiomyopathy–Associated Desmosomal Mutation Carriers. J. Am. Coll. Cardiol. 2013;62:1761–1769.

4. Bhonsale A, te Riele ASJM, Kamel IR, et al. Yield of Serial Evaluation in At-Risk Family Members of Patients With ARVD/C. J. Am. Coll. Cardiol. 2014;64:293–301.

5. Bhonsale A, Groeneweg JA, James CA, et al. Impact of genotype on clinical course in arrhythmogenic right ventricular dysplasia/cardiomyopathy-associated mutation carriers. Eur. Heart J. 2015;36:847–855.

6. Kelly MA, Caleshu C, Morales A, et al. Adaptation and validation of the ACMG/AMP variant classification framework for MYH7-associated inherited cardiomyopathies: recommendations by ClinGen’s Inherited Cardiomyopathy Expert Panel. Genet. Med. 2018;20:351–359.

7. Richards S, Aziz N, Bale S, et al. Standards and guidelines for the interpretation of sequence variants: a joint consensus recommendation of the American College of Medical Genetics and Genomics and the Association for Molecular Pathology. Genet. Med. 2015;17:405–423.

Tables

Additional file 1: Table S1. Table comparing the demographic characteristics between patients who were included (n=73) as well as those who were excluded from our study (n=29). As displayed in the table, there is no statistically significant difference in demographic characteristics between the two groups.

|  | **Study Cohort (n=73)** | **Excluded Participants (n=29)** | **p-value** |
| --- | --- | --- | --- |
| Age, *in years* | 34.2 ±13.5 | 34.3 ±15.9 | 0.97 |
| Male, *(%)* | 37 (50.7) | 16 (55.1) | 0.69 |
| TFC, *median [IQR]* | 6 [5-7] | 6 [5-7] | 0.46 |
| Proband, *(%)* | 41 (56.2) | 16 (55.1) | 0.92 |

| **Patient ID** | **Pedigree** | **Gene** | **DNA Change** | **Amino Acid Change** |
| --- | --- | --- | --- | --- |
| 1 | Proband | PKP2 | c.2197_2202delCACACCinsG | p.(His733Alafs*8) |
| 2 | Family Member | PKP2 | c.235C>T | p.(Arg79*) |
| 4 | Family Member | PKP2 | c.1237C>T | p.(Arg413*) |
| 5 | Proband | PKP2 | c.1803delC | p.(Glu601fs*55) |
| 7 | Family Member | PKP2 | c.2509delA | p.(Ser837Valfs*94) |
| 9 | Proband | PKP2 | c.2197_2202delCACACCinsG | p.(His733Alafs*8) |
| 11 | Proband | PKP2 | c.235C>T | p.(Arg79*) |
| 12 | Family Member | PKP2 | c.2489+1G>A | mutant splice product |
| 13 | Family Member | PKP2 | c.968_971delAGGC | p.(Gln323fs) |
| 14 | Family Member | PKP2 | c.2509delA | p.(Ser837Valfs*94) |
| 15 | Family Member | PKP2 | c.968_971delAGGC | p.(Gln323fs) |
| 16 | Family Member | PKP2 | c.2146-1G>C | mutant splice product |
| 18 | Proband | PKP2 | c.1613G>A | p.(Trp538*) |
| 27 | Family Member | PKP2 | c.235C>T | p.(Arg79*) |
| 28 | Proband | PKP2 | c.1759delG | p.(Ser587fs*69) |
| 30 | Family Member | PKP2 | c.1844C>T | p.(Ser615Phe) |
| 32 | Family Member | PKP2 | c.2013delC | p.(Lys672ArgfsX12) |
| 33 | Family Member | PKP2 | c.1237C>T | p.(Arg413*) |
| 37 | Family Member | PKP2 | c.2509delA | p.(Ser837Valfs*94) |
| 39 | Family Member | PKP2 | c.2169_2172dupAGTT | p.(Val7255Serfs*19) |
| 41 | Family Member | PKP2 | c.2489+1G>A | mutant splice product |
| 44 | Family Member | PKP2/DSP* | c.2197_2202delCACACCinsG; c.del6p24.1p25 | p.(His733Alafs*8); multi-gene deletion |
| 46 | Proband | DSP | c.151C>T | p.(Gln51*) |
| 48 | Proband | DSG2/DSG2* | c.1038_1040delGAA; c.523+2T>C | p.(Lys346del); mutant splice product |
| 49 | Family Member | PKP2 | c.2013delC | p.(Lys672ArgfsX12) |
| 51 | Proband | PKP2 | c.148_151delACAG | p.(Thr50Serfs*61) |
| 55 | Proband | PKP2 | c.2197_2202delCACACCinsG | p.(His733Alafs*8) |
| 56 | Proband | PKP2 | c.1760delT | p.(Val587fs*69) |
| 58 | Proband | PKP2 | c.1613G>A | p.(Trp538*) |
| 60 | Proband | DSG | c.136C>T | p.(Arg46Trp) |
| 61 | Proband | PKP2 | c.148_151delACAG | p.(Thr50Serfs*61) |
| 64 | Family Member | PKP2 | c.2146-1G>C | mutant splice product |
| 68 | Family Member | PLN | c.40_42delAGA | p.(Arg14del) |
| 70 | Proband | PKP2 | c.235C>T | p.(Arg79*) |
| 73 | Proband | PKP2 | c.2146-1G>C | mutant splice product |

Additional file 1: Table S2. Table summarizing genetic characteristics of study participants with ARVC/D associated mutations.

**Compound mutation.*

Additional file 1: Table S3 - Table summarizing clinical and CMR characteristics of ARVC/D study participants with isolated LV involvement. ARVC/D: Arrhythmogenic right ventricular cardiomyopathy/dysplasia, LGE: late-gadolinium enhancement, LV: left ventricular, RV: right ventricular, TFC: task force criteria.

| **Clinical Characteristics** | **Patient 46** | **Patient 68** |
| --- | --- | --- |
| 2010 Task Force Criteria met for diagnosis of ARVC | - Major repolarization abnormalities criteria (negative T-wave in V1-3) - Major and minor depolarization/conduction abnormalities criteria (epsilon wave in V1-3, terminal activation duration of QRS >55ms in V1-3) - Minor arrhythmia criteria (>500 PVCs/24h) - Major family history criteria (ARVD/C-associated pathogenic mutation). | - Minor repolarization abnormalities criteria (T-wave inversion in V4-6) - Minor arrhythmia criteria (>500 PVCs/24h) - Major and minor family history criteria (ARVD/C-associated pathogenic mutation, history of ARVC/D in a first-degree relative in whom it is not possible to determine whether the family member meets current TFC). |
| 2010 TFC Score | 7 | 5 |
| Age | 48 | 39 |
| Gender | Female | Female |
| Proband | Proband | Family Member |
| Mutation | DSP(c.151C>T) | PLN(c.40_42delAGA) |
| RV Ejection Fraction | 51% | 47% |
| LV Ejection Fraction | 43% | 57% |
| LV Morpho-Functional Abnormalities, *if present* | LV wall motion abnormalities | None |
| LV Tissue Abnormalities | Mid- and Sub-epicardial LGE (AHA segments 1-10, 12, 16) in both patchy and linear patterns | Mid- and Sub-epicardial LGE (AHA segments 5, 11, 15, 16) in both patchy and linear patterns |
| VT During Follow-up | No | No |

Additional file 1: Table S4- Table comparing baseline demographic, clinical and genetic characteristics as well as arrhythmic outcomes between study participants with normal left ventricular EF with vs. without LV tissue abnormalities. CMR: cardiac magnetic resonance imaging, EF: ejection fraction, LV: left ventricular, TFC: task force criteria, VT: ventricular tachycardia.

|  | **Participants with normal LVEF and no LV tissue abnormalities (n=36)** | **Participants with normal LVEF and LV tissue abnormalities (n=19)** | **p-value** |
| --- | --- | --- | --- |
| Age, *in yrs* | 34.8**±**15.7 | 35.6**±**12.4 | 0.85 |
| Male Gender, *n(%)* | 16 (44.4) | 10 (52.6) | 0.58 |
| TFC, *median [IQR]* | 5 (4-6.5) | 6 (5-8) | 0.011 |
| Proband, *n(%)* | 14 (38.9) | 14 (73.7) | 0.023 |
| Gene Positive, *n(%)* | 17 (47.2) | 9 (47.4) | 1.00 |
| VT Prior to CMR, *n(%)* | 12 (33.3) | 9 (47.4) | 0.39 |
| VT During Follow-up, *n(%)* | 15 (41.7) | 12 (63.2) | 0.16 |

**Online Appendix**

Figures

Additional file 1: Figure S1- Kaplan-Meier Survival function curve comparing frequency incident life-threatening ventricular arrhythmias in ARVC/D patients with preserved left ventricular EF with and without LV tissue abnormalities on CMR (LGE or fat infiltration). P-value of log-rank test comparing the two curves was 0.08.

**
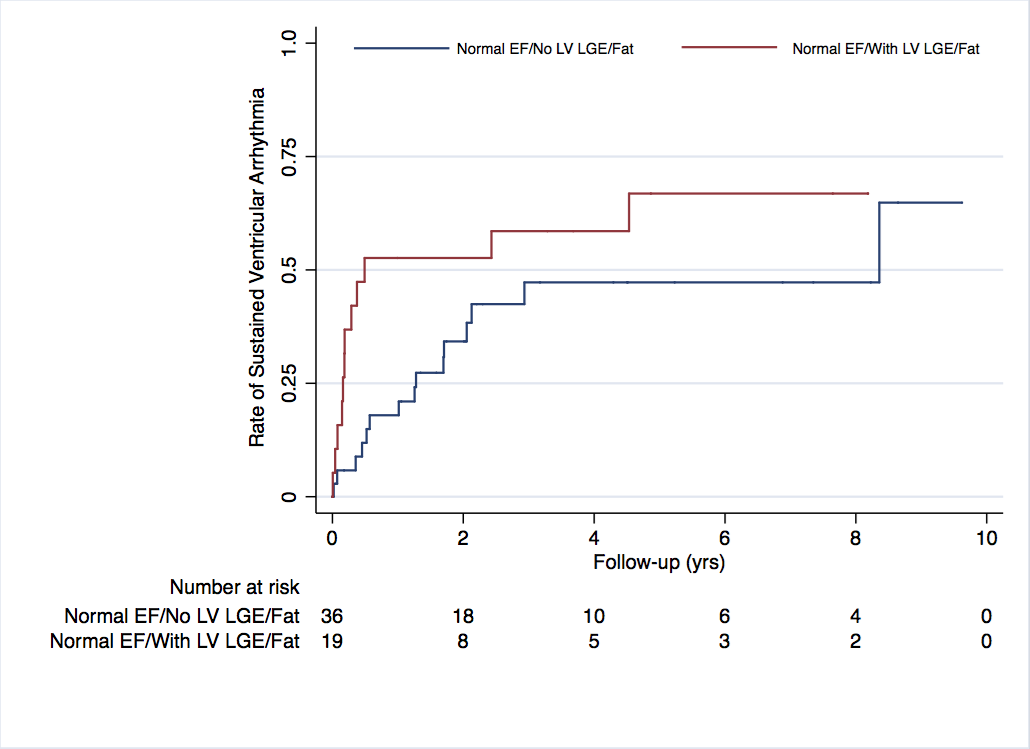
**
